# Additive manufacturing of poly (lactic acid)/hydroxyapatite/carbon nanotubes biocomposites for fibroblast cell proliferation

**DOI:** 10.1038/s41598-023-47413-0

**Published:** 2023-11-21

**Authors:** Francilene da Luz Belo, Esleane Vilela Vasconcelos, Miriane Alexandrino Pinheiro, Damares da Cruz Barbosa Nascimento, Marcele Fonseca Passos, Alisson Clay Rios da Silva, Marcos Allan Leite dos Reis, Sérgio Neves Monteiro, Rebecca Thereza Silva Santa Brígida, Ana Paula Drummond Rodrigues, Verônica Scarpini Candido

**Affiliations:** 1 Engineering of Natural Resources of the Amazon Program, Federal University of Pará–UFPA, Belém, Brazil; 2 Materials Science and Engineering Program, Federal University of Pará-UFPA, Belém, Brazil; 3 Materials Science Program, Military Institute of Engineering—IME, Rio de Janeiro, Brazil; 4 Electron Microscopy Laboratory, Evandro Chagas Institute-IEC, Belém, Brazil

**Keywords:** Engineering, Materials science

## Abstract

Bone tissue is one of the most important in the human body. In this study, *scaffolds* of poly (lactic acid) PLA reinforced with hydroxyapatite (HA) and carbon nanotubes (CNT) were manufactured, evaluating their mechanical and biological properties. HA was synthesized by wet method and characterized by X-ray diffraction (XRD), Fourier transform infrared spectroscopy (FTIR), and scanning electron microscopy (SEM). The *scaffolds* were produced using additive manufacturing and characterized by optical microscopy, SEM, thermogravimetric analysis (TGA), Raman spectroscopy and biological tests. The SEM results showed that the PLA surface was affected by the incorporation of CNT. TG showed that the incorporation of HA into the polymer matrix compromised the thermal stability of PLA. On the other hand, the incorporation of CNT to the polymer and the impregnation with HA on the surface by thermal effect increased the stability of PLA/CNT *scaffolds*. Raman spectra indicated that HA impregnation on the surface did not modify the polymer or the ceramic. In the compression tests, PLA and PLA/CNT *scaffolds* displayed the best compressive strength. In the biological tests, more than 85% of the cells remained viable after 48 h of incubation with all tested *scaffolds* and groups with CNT in the composition disclosing the best results.

## Introduction

Bone tissues is one of the most important tissues in the human body, being composed of several elements, including collagen fibers and hydroxyapatite (HA) crystals, responsible for the strength and hardness of the bone^1,2^. As the human body ages, many changes take place, which can compromise bone structure and interfere with its functions^3,4^. Additionally, external events such as traumatic occurrences may also compromise this structure by causing fractures that, depending on the severity, can lead to temporary or permanent disability and, in more severe cases, even death^4,5^. Trauma is the most common cause of death among ages 1–44 and accounts for about 80% of deaths among teenagers, all over of the world^4–6^. Furthermore, in United States of America, 60% of childhood deaths are by virtue to trauma, still being the seventh leading cause of death in the elderly^5,6^.

Bone fracture is usually the most common event in traumatic occurrences. Therefore, the use of biomaterials becomes an interesting alternative in the development of new materials to remedy the loss of bone tissue. Biomaterials comprise a representative fraction of products used in healthcare. Between them, we can mention implantable materials, and artificial organs, as well as many others^7,8^. Among the biomaterials, bioceramics have been successfully used in the human body for many years, with an essential role in orthopedics and dentistry. It and can be classified according to their interaction with the tissue^7,9^. Among bioceramics, HA stands out as the most used for clinical purposes, mainly because it is the major component of the mineral phase of the bones and teeth^7^. It is a very important mineral for society and plays a prominent role in the repair of bone defects, being an attractive choice as a biomaterial due to its similarity with the bone mineral component^10^. Additionally, HA significantly improves the bioactivity, osseointegration, catalytic activity, and adsorption capacity of bone repair materials^11,12^.

With great regenerative potential among all other human body tissues, bones are considered to be suitable candidates for the technique of tissue engineering^3,9,13^. In this type of engineering technique, various materials are used as *scaffolds*, that play a fundamental role in the process of bone repair. *Scaffolds* are synthetic bone substitutes that must have properties compatible with their application, including biocompatibility, bioactivity, osteoconduction, osteoinduction and biodegradability, favoring the regeneration of bone tissue. They can be produced as biocomposites from HA and ceramics incorporated into synthetic or natural polymers or a mixture of both^14–16^.

Among synthetic polymers used in the production of biocomposites, poly (lactic acid) (PLA) has been extensively studied for the biocompatibility, biodegrading into non-toxic components, with a controllable rate of degradation after introduction into the human body; an important characteristic in the biomaterials^2,13,14^. Depending on the stereochemical composition and molecular weight, the tensile strength of PLA is approximately 50–70 MPa and the elastic modulus between 3.0 and 4.0 GPa^9,13^. Therefore, PLA is an excellent option for making *scaffolds*, biomaterials that need to suffer gradual degradation during the process of bone regeneration^7,9,14^. Furthermore, the mechanical properties of PLA *scaffolds* can be improved with the incorporation of carbon nanotubes (CNT) into the polymeric matrix^2^. From a structural standpoint, CNTs can be divided into single-walled CNTs (SWCNTs) and multi-walled CNTs (MWCNTs), where MWCNTs have lower toxicity than SWCNTs, considered one of the most promising applications of nanotechnology for the production of biomaterial^17,18^. The benefits of CNT addition into biomaterials have been studied in recent years, and many researches show improvement in the resistance and other properties of biomaterials^2,7,10,17,18^. CNTs have excellent mechanical properties, which make them great candidates for the production of high-performance biocomposites, contributing to accelerate bone growth and promote proliferation of osteoblastic cells, which speeds up the bone tissue regeneration^2,18,19^.

In this context, and in an attempt to improve the treatment of patients with injuries resulting in damage to body parts, tissue engineering presents itself as an emerging field to develop biological substitutes of tissues or organs, using additive manufacturing, also known as 3D printing as a transformative tool for biomedical applications^20,21^. In the additive manufacturing technology, a three-dimensional model is created by successive layers of material. In the context of orthopedic surgeries, it is a strategic alternative to conventional reconstructive surgery methods, intending to promote bone tissue regeneration^2,13,20,22^. Therefore, the objective of this study was to produce, by 3D printing, *scaffolds* made of PLA reinforced with HA and CNT, for use as biomaterials. Despite the literature showing publications associating HA and PLA for 3D printing of *scaffolds* for the regeneration of bone tissue, so far, no study has been found associating HA and CNT with the PLA to produce biocomposites as proposed in this study. Furthermore, the influence of internal spacing on the properties of PLA/HA/CNT *scaffold*s was not yet evidenced. It is clear that the combination of PLA, HA and CNT, associated with geometry and internal spacing, can promote greater biocompatibility and improve the properties of the *scaffolds* produced by 3D printing.

## Results and discussion

### Production and characterization of the materials used to produce the *scaffolds*

#### Hydroxyapatite powder X-ray diffraction

**Figure 1:**
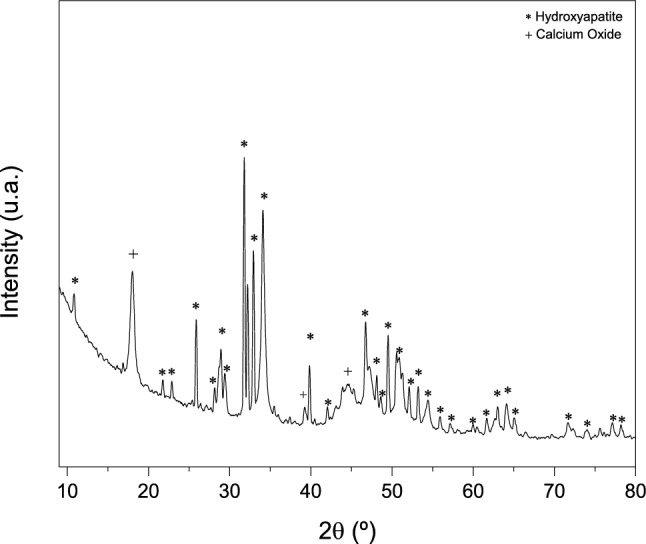
X-ray diffractogram of HA powder synthesized by acid–base reaction and calcined at 1,000°C.

The x-ray diffraction (XRD) pattern of the HA powder sample is shown in Fig. 1. XRD patterns DISPLAY characteristic HA peaks as the predominant phase in the sample, according to ICSD 026205. Comparing the peaks with the standards found in the literature, it clearly shows the effectiveness of the synthesis and the crystallinity of the material obtained^23,24^. The peak intensities reveals that the calcination temperature promoted mineral crystallinity. Samples of HA sintered at 1000 °C show greater crystallinity^12,25^. Furthermore, the increase in temperature can also interfere with the size of the grains and promote the production of nanometric HA^14,24^. In addition to the HA, there was also the formation of calcium oxide, according to ICSD 060199, which may have been generated as a result of the calcination process. Indeed, synthetic calcium hydroxide was used during the synthesis of HA. As indicated^24,26^, calcium oxide arises from the high calcination temperatures that lead to increased crystallinity and the formation of non-stoichiometric HA, deficient in calcium. This feature is not undesirable since non-stoichiometric HA does not change the biocompatibility, as it is similar to biological HA^26,27^.

The Reitveld refinement of HA powder is shown in Table 1. The Rietveld refinement takes into account the reliability parameters obtained by the weighted profile (R_wp_), the expected profile (R_exp_), pattern profile (R_p_) and Bragg factor (R_Bragg_). The quality of fit (χ^2^) is obtained by the ratio of R_wp_ e R_exp_. O R_wp_ is what best represents the progress of refinement, so values in the order of 2–20% are considered successful. The quality of fit (χ2) must have standards that determine the efficiency of the refinement, which can vary between 1 (considered perfect) and 5 (reliable refinements). The statistical indices of the refinement (R_Bragg_, R_wp_, R_exp_, and χ^2^) were considered satisfactory, as the overall index χ2 = (R_wp_/R_exp_)^2^ was less than 5. Furthermore, it can be seen that the quantity of HA is equal to 98.47% and CaO is 1.53%, showing that, despite the calcination temperature, there is a preferential formation of HA.

**Table 1:** Reitveld refinement of HA powder.

Sample	Component (wt%)		Indices				
	HA	CaO	R_wp_	R_exp_	R_p_	R_Bragg_	χ^2^
1.0HA1000	98.47	1.53	4.22	3.85	3.30	3.55	1.15

*R*_*wp*_ the weighted profile, *R*_*exp*_ the expected profile, *R*_*p*_ profile, *R*_*Bragg*_ Bragg factor, *χ*^*2*^ the goodness of fit.

#### Fourier transform infrared spectroscopy (FTIR) of hydroxyapatite powder

**Figure 2:**
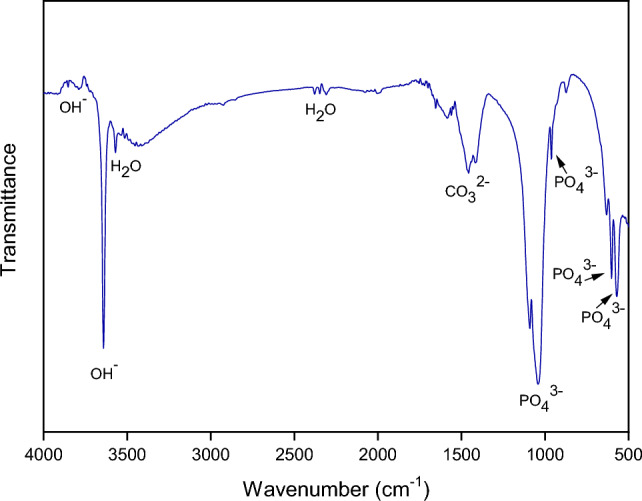
FTIR of HA powder synthesized by acid–base reaction at 1000°C.

Figure 2 presents the image of the infrared spectrum, and Table 2 shows the functional groups and wavelengths of the synthesized HA powder. The presence of absorption bands is observed in 3788, 3640, 3570, 1457, 1040, 960, 600 and 570 cm^−1^. The bands around 3640 and 3788 cm^−1^ are related to the stretching vibrations of the OH group present in the HA^28,29^. According to Kumar et al.^30^, the band 1457 cm^−1^ is attributed to asymmetric elongation of the carbonate group. The peaks in the band around 1400 cm^−1^ are the result of the adsorption of carbon dioxide present in the atmospheric air, causing an enlargement of the peak area due to their overlapping^12,28,30^. The absorption bands between 1040 up to 570 cm^−1^ are related to the asymmetric deformation of the phosphate groups^27,30^, similar to the characteristics of HA.

**Table 2:** Functional groups and wavenumbers of HA powder.

Functional groups	Wavenumbers (cm^−1^)
OH–	3788
OH–	3640
CO_3_^2−^	1457
PO_4_^3−^	1040–960–600–570

#### Scanning electron microscopy (SEM) of hydroxyapatite powder

**Figure 3:**
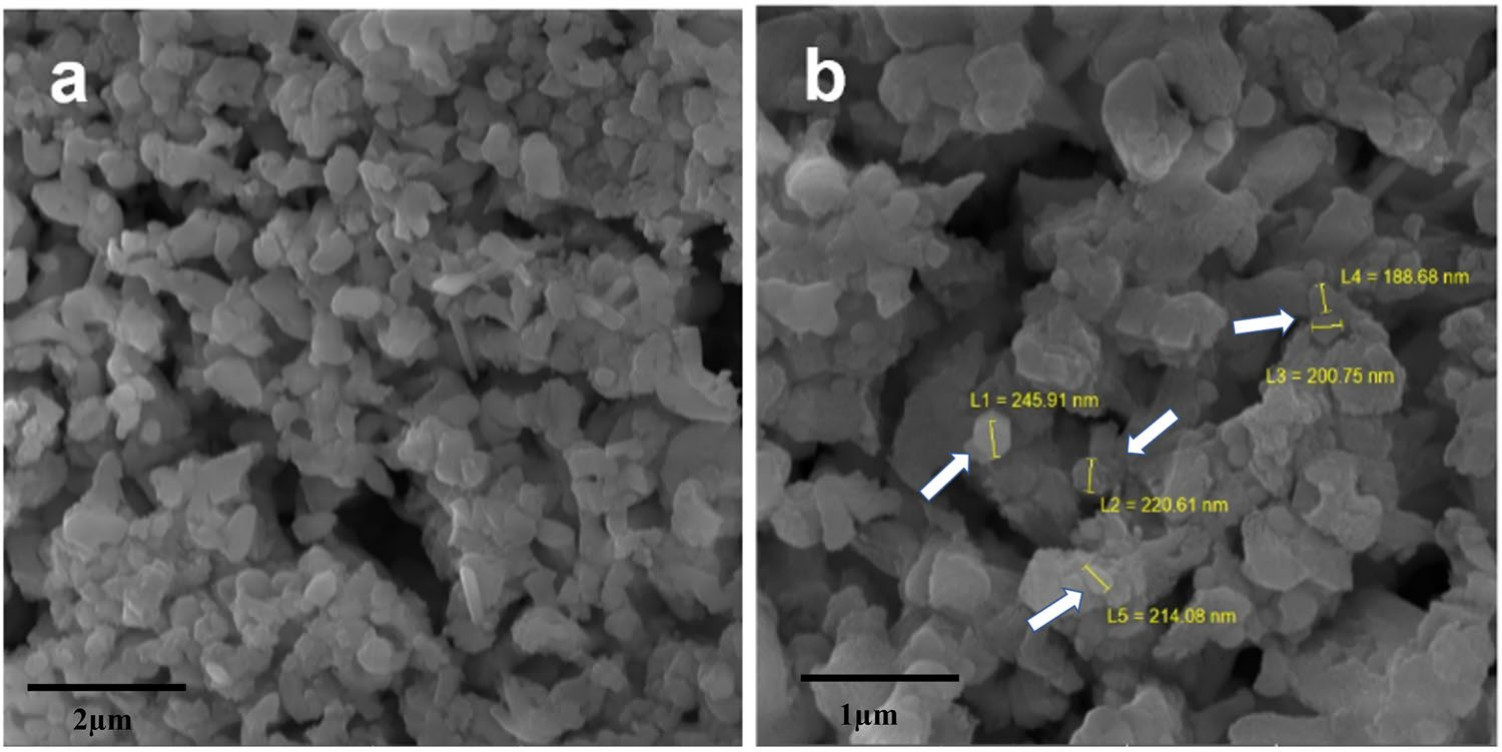
Micrograph of hydroxyapatite powder synthesized at 1000°C by acid–base reaction. (**a**) 27.7 kx magnification; (**b**) 55.4 kx magnification.

**Figure 4:**
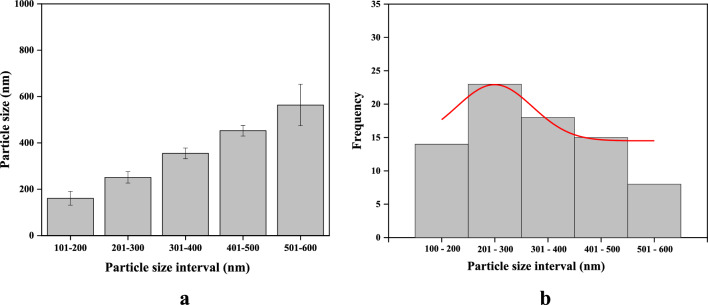
Particle size of HA powder. (**a**) Particle size vs particle size interval and (**b**) Frequency of particle size vs particle size interval.

Figure 3 shows the micrographs of the HA powder, revealing the formation of small asymmetrical crystals that confirm the formation of the “hydroxyapatite phase”. The morphology of the HA powder shows an agglomeration of small grains scattered on the surface. The variations in the grain size and morphology can help to differentiate and understand the physical–chemical and biomechanical aspects of a biomaterial^28,31–33^. As shown in Fig. 3b, clusters similar to prismatic crystallites can be seen with hexagonal basal sections characteristic of HA, indicating the formation of the “hydroxyapatite phase”^1,8,12^. It is also possible to observe an average size of the grains ranging approximately from 188.68 to 245.21 nm. Such a characteristic may demonstrate the existence of a nanomaterial^12,24,31^. Thereby, evidence has proven that nanometric-scale particles can accelerate osteoconduction and osteointegration processes in bone tissues^10,24,30^. Figure 4 shows the particle size distribution of HA powder. In Fig. 4a it can be noted that HA has average particle sizes that vary between 501 and 600 nm, but smaller particles are also observed, with sizes from 100 to 300 nm. In Fig. 4b, it can be noted, despite the grains having an average size between 501 and 600 nm, that there is a greater number of grains in the range comprising 201 and 300 nm.

#### Energy dispersive spectroscopy (EDS) of hydroxyapatite powder

**Figure 5:**
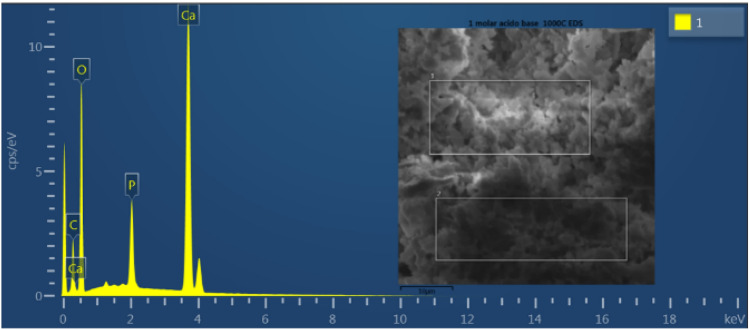
EDS spectrum of hydroxyapatite powder.

The EDS mapping of the HA powder sample presented in Fig. 5 is a qualitative estimate, as it does not represent the entire sample. The results are compatible with the XRD. It is also observed, in Table 3, that the HA formed has a Ca/P ratio equal to 1.50, which suggests it is deficient in calcium. This pattern may be associated with the calcination temperature used, which favors the formation of non-stoichiometric HA. Furthermore, it can be suggested that the formation of this non-stoichiometric HA may have been favored by the formation of CaO, as observed in the XRD. The Ca/P ratio of stoichiometric material is 1.67. However, stable compositions can also show an extension of the Ca/P ratio to approximately 1.50, assuming there is no ionic substitution in the apatite structure^34^. Despite noting a non-stoichiometric HA, the chemical composition of HA (Ca, P, O, H), in the sample analyzed, reveals a significant variation in the distribution of minerals dispersed on the surface is observed, with a higher concentration of calcium in relation to phosphorus, which is in line with what is theoretically expected for HA^24,28,32^. It should be noted that these elements are important for the biocompatibility of the material under study, as it can induce cell colonization, contributing to the replacement of the biocomposite by bone tissue during the process of cell regeneration^14,24,31^.

**Table 3:** Percentage of chemical elements: calcium (Ca); phosphorus (P) and oxygen (O).

Elements						Ca/P
Weight %			Atomic %			
Ca	P	O	Ca	P	O	
28.48	14.65	56.88	15.00	9.98	75.03	1.50

#### 3D printing of *scaffolds* and the impregnation with HA powder on the surface of the *scaffolds*

**Figure 6:**
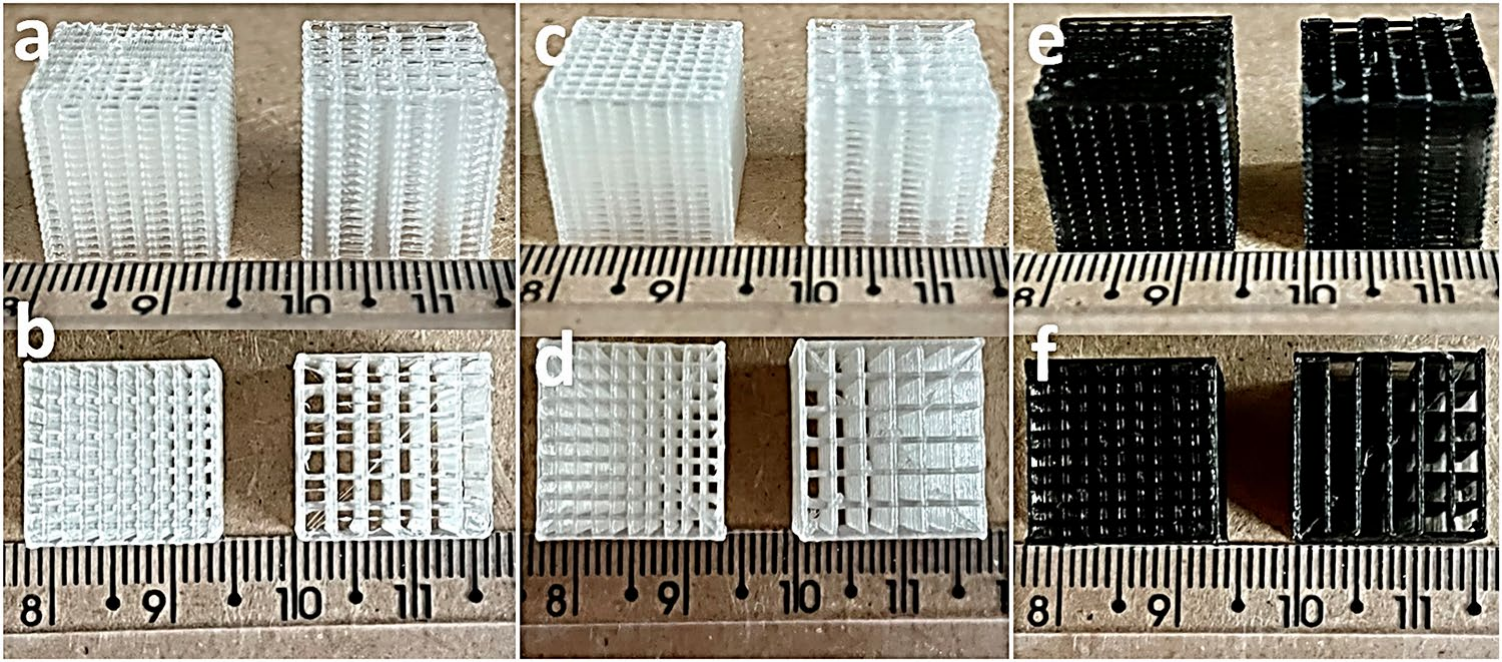
Perspective and top view, respectively. 3D printing of 1 and 2 mm squarebar boxes *scaffold*s. (**a**,**b**) pure PLA; (**c**,**d**) PLA/HA; (**e**,**f**) PLA/CNT.

Figure 6 shows the pure PLA, PLA/HA and PLA/CNT *scaffolds* 3D printed using additive manufacturing, by the fused depositions modeling (FMD), with squarebar boxes opening varying from 1.0 and 2.0 mm. The stability of PLA *scaffolds* can be affected, depending on the gaps between the *scaffolds* filaments^8,17,35,36^. Different openings in the squarebar boxes can also interfere with the compressive strength of the material, which needs to be comparable with the compressive strength of cancellous bone, which can vary from 4 to 15 MPa^3,9,14,35^. Variations in the wall thickness of the pure PLA filament after coating with PLA/HA and PLA/CNT solutions are described in Table 4. Furthermore, after the 3D printing process and impregnation with HA powder on the surface of the *scaffolds* by chemical and thermal effect, a variation in mass was observed for the impregnated samples, as shown in Table 5. This mass gain is attributed to the adhesion of the HA powder to the surface of the *scaffold*s, thus showing that the impregnation process was effective. Moreover, it is noted that the samples with squarebar boxes opening with 1.0 mm, presented, on average, a greater mass gain than the *scaffold*s with opening of 2.0 mm. This factor might be associated with the smaller distance between the walls, which allowed better aggregation of the HA powder on the PLA surface.

**Table 4:** Thickness variation of pure PLA filament after coating with PLA/HA and PLA/CNT solution.

Filament	Thickness (mm)
Pure PLA	1.75
PLA/HA	1.85
PLA/CNT	1.78

**Table 5:** Mass gain after HA impregnation by themal effect and chemical effect on the surface of *scaffolds*.

Sample	Squarebar boxes (mm)	Initial mass (M_I_)		Final dough (Fg)		Variation	
		Thermal (g)	Chemical (g)	Thermal (g)	Chemical (g)	Thermal (g)	Chemical (g)
Pure PLA	1.0	0.03	0.04	0.03	0.04	0.001	0.003
	2.0	0.04	0.03	0.04	0.03	0.001	0.001
PLA/HA	1.0	0.08	0.06	0.08	0.08	0.001	0.004
	2.0	0.03	0.04	0.03	0.04	0.001	0.002
PLA/CNT	1.0	0.07	0.06	0.07	0.07	0.002	0.005
	2.0	0.04	0.04	0.04	0.04	0.002	0.002

#### Optical microscopy and scanning electron microscopy (SEM) of the *scaffolds*

**Figure 7:**
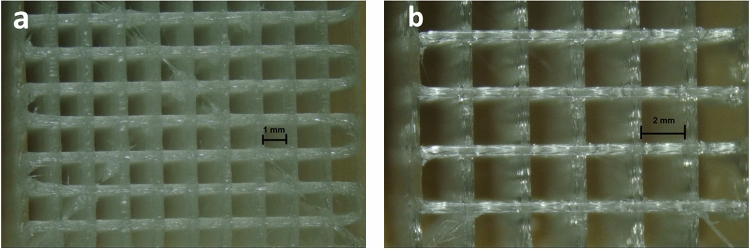
Top view. Optical microscopy of *scaffold*s with 1 mm squarebar boxes (**a**) and *scaffold*s with 2 mm squarebar boxes (**b**). 10 × magnification.

**Figure 8:**
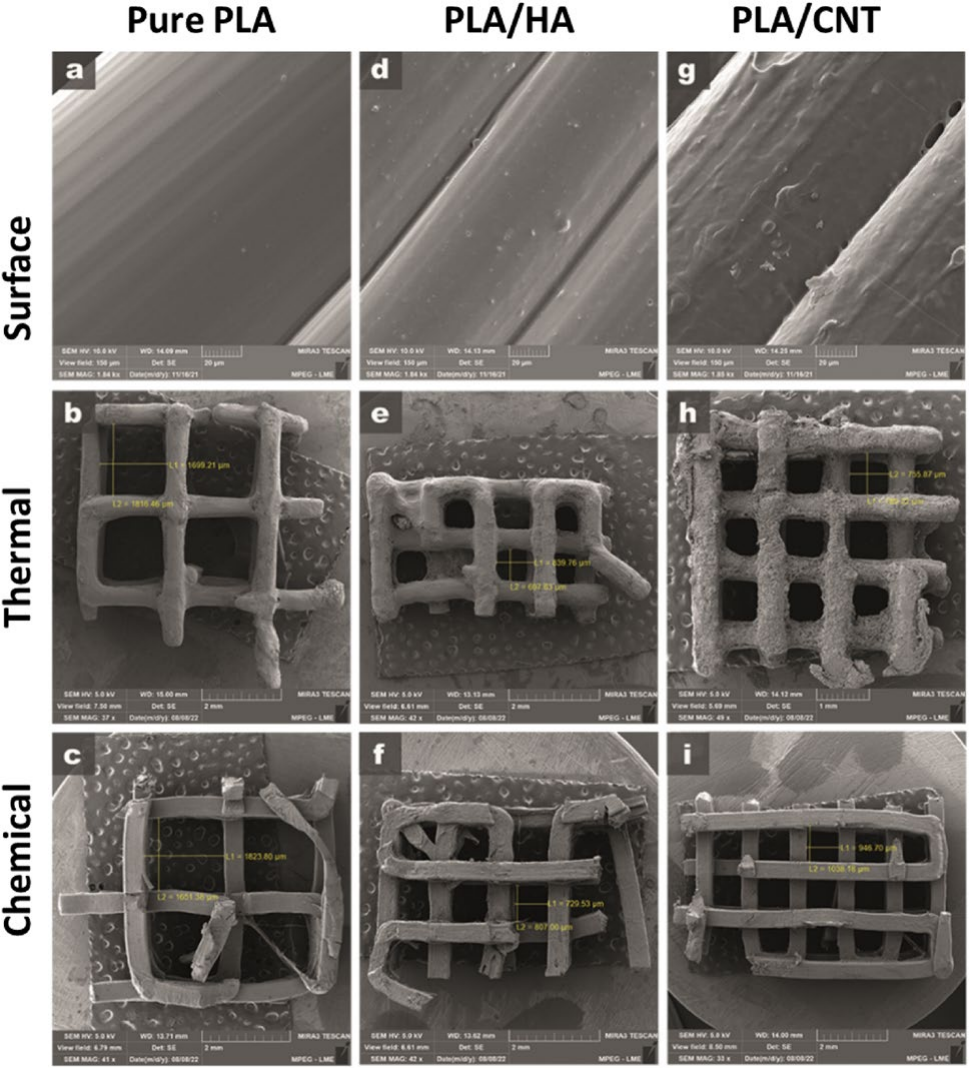
SEM of *scaffold*s produced under different conditions. (**a**–**c**) pure PLA; (**d**,**e**) PLA/HA; (**g**–**i**) PLA/CNT.

Figure 7 presents the optical microscopy of pure PLA, PLA/HA and PLA/CNT *scaffolds* manufactured by 3D printing using the FDM technique, with variations in 1.0 and 2.0 mm squarebar boxes. Regular spacings are observed, with sizes varying between 1.0 and 2.0 mm. This variation can interfere with the mechanical properties of *scaffolds*^36,37^. Figure 8 shows the micrographs of pure PLA (a–c), PLA/HA (d–f), and PLA/CNT (g–i) *scaffolds*. In the pure PLA samples (Fig. 8a) a smooth surface is observed, whereas PLA/HA (Fig. 8d) and PLA/CNT (Fig. 8g) samples demonstrate a rough surface. It is also related in studies that the addition of CNT significantly affects the surface of PLA, making it rougher compared to the HA-containing sample^2,17,37^.

#### Thermogravimetric analysis (TGA) of *scaffolds*

**Figure 9:**
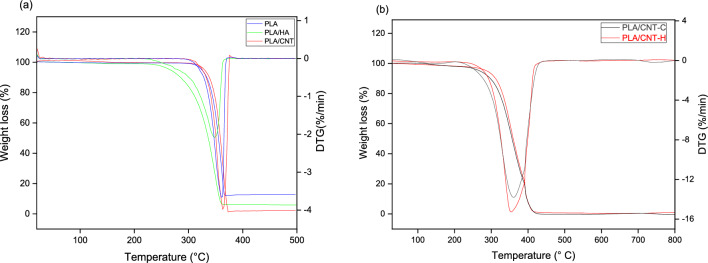
TGA and DTG were obtained for (**a**) pure PLA; PLA/HA; PLA/CNT without treatment and (**b**) PLA/CNT impregnated with HA on the surface by thermal (H) and chemical effect (C).

Figure 9 shows the TGA/DTG curves for PLA, PLA/HA and PLA/CNT *scaffolds*, (9a) without impregnation and (9b) with impregnation of HA on the surface. Pure PLA displays the beginning of thermal decomposition at 296.67 °C, while the PLA/HA exhibits the beginning of thermal decomposition at a lower temperature, 226.69 °C. It is clear that the addition of HA to the PLA matrix interferes with the thermal stability of the polymer, because the HA is more unstable at high temperatures. According to^16,38,39^, a temperature around 200 °C can promote mass loss in pure HA, which can be attributed to the removal of adsorbed water at this temperature. According to a study by Mustafov, Sen and Seydibeyoglu^38^, a HA reinforced polymeric matrix may exhibit slow mass loss between 279 and 396 °C, which confirms the result of this study for the PLA/HA *scaffold*. Furthermore, PLA/CNT already shows the beginning of thermal decomposition at 299.90 °C, demonstrating greater stability among the studied *scaffolds*. This result can be attributed to the thermal stability of the raw CNT, which is usually above 600 °C^40,41^. In this study, it was analyzed that the addition of CNT to the polymeric matrix demonstrated a positive effect on the thermal stability of the composite. Another important factor to be considered for this result may be attributed to the interaction between PLA and CNT. In this case, as the CNTs restrict the movement of the PLA chains, inhibiting chain fusion during the degradation process, the TG of PLA/CNT is slightly higher than pure PLA^40,42^.

Considering the samples impregnated with HA on the surface (Fig. 9b), for PLA/CNT surface-impregnated with HA by chemical effect (PLA/CNT-C) e PLA/CNT impregnated with HA by thermal effect (PLA/CNT-H) the beginning of thermal decomposition is observed at 216.58 and 250.31 °C, respectively, demonstrating that HA promoted a decrease in thermal stability, when compared to PLA/CNT samples. Furthermore, it is observed that these samples showed greater mass loss when compared to the other compositions studied and that the chemical effect decreased the thermal stability of the *scaffold*s. This is shows that both acetone and HA negatively influence the stability of the material and to demonstrate that the best method of coating with HA is the thermal effect. Thermal degradation parameters are summarized in Table 6.

**Table 6:** TGA and DTG degradation parameters of pure PLA, PLA/HA and PLA/CNT samples without impregnation and impregnated by thermal effect (H) and chemical effect (C).

Sample	T_onset_ (°C)	T_endset_ (°C)	DTG peak (°C)	Mass variation (%)
Pure PLA	296.67	378.40	361.16	95.18
PLA/HA	226.69	365.75	347.07	92.68
PLA/CNT	299.90	381.63	363.10	93.24
PLA/CNT-C	216.58	436.75	353.90	97.87
PLA/CNT-H	250.31	448.61	359.18	96.71

### Raman spectroscopy

**Figure 10:**
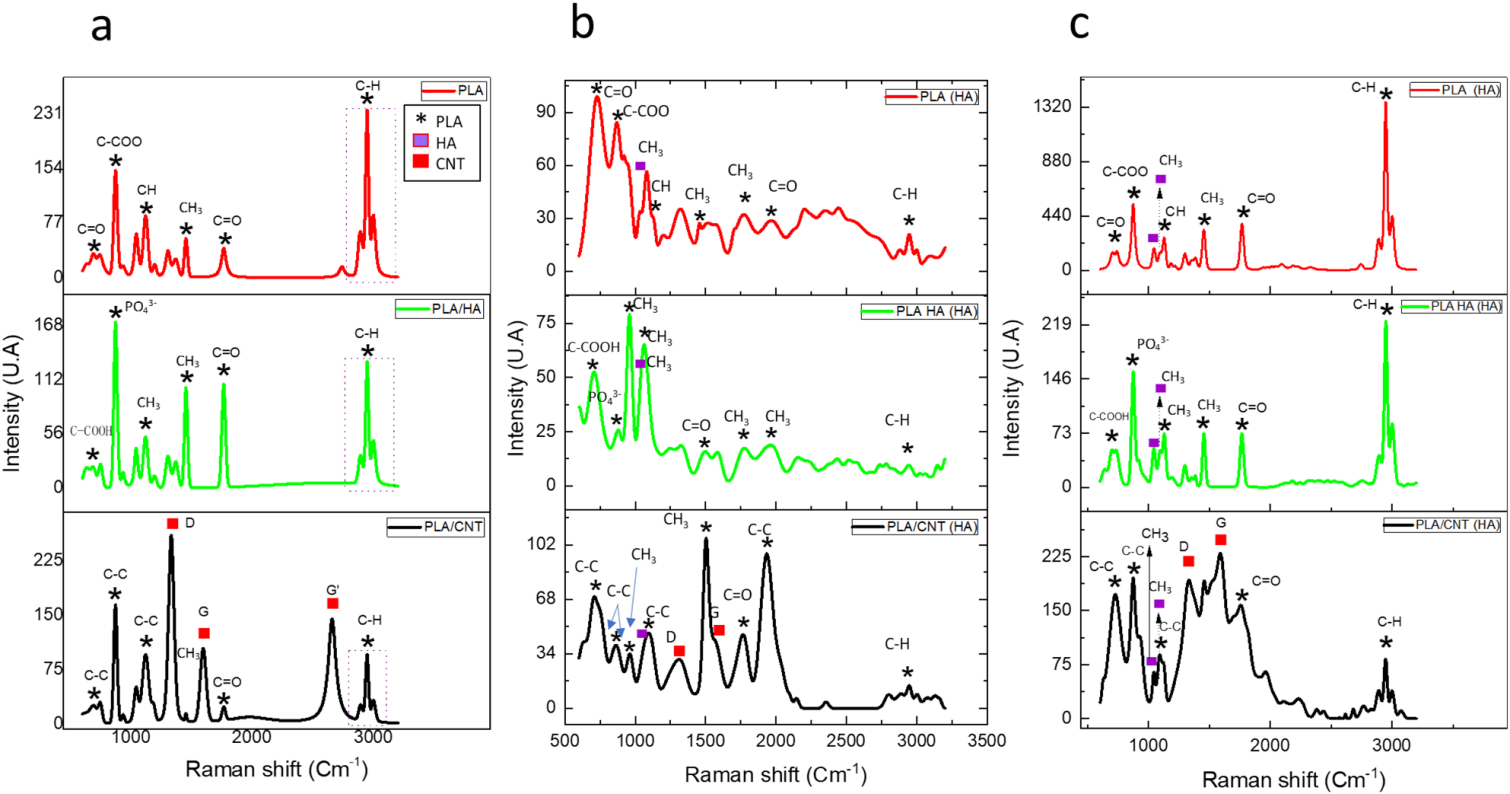
Raman spectroscopy of PLA, PLA/HA, PLA/CNT *scaffold*s. (**a**) *Scaffold*s without impregnation; (**b**) impregnated by thermal effect; (**c**) impregnated by chemical effect.

Figure 10 shows the vibrational spectra (Raman) for pure PLA, PLA/HA and PLA/CNT, (Fig. 10a) without impregnation, (Fig. 10b) with HA impregnation on the material surface by H-thermal effect and (Fig. 10c) by C-chemical effect. It is clear that the wavenumbers referring to PLA remained apparent in all spectra for all materials, observed from around 632 to 3000 cm^−1^. However, in the sample of the PLA impregnated with HA on the surface through thermal effect (Fig. 10b), a shift of the peaks to the left is observed, which can be attributed to asymmetrical and symmetrical stretching vibrations between the C-H bonds^43–45^. As for the vibrations referring to HA, these begin to be seen around 962–1085 cm^−1^. These bands can be attributed to the PO_4_^3−^ elongation vibrations that appear around 960 cm^−1^ as already described in the literature^43,44^. Concerning the PLA/HA composite (Fig. 10a), one notices that the first vibrations also appear around 632 cm^−1^. However, by undergoing thermal effect impregnation (Fig. 10b), the first vibrations are observed at around 601 cm^−1^ and with the chemical effect (Fig. 10c), it is observed at around 629 cm^−1^. These graphs may indicate that thermal and chemical processes do not considerably modify the PLA polymer or the HA ceramic. For PLA/CNT composite (Fig. 10a), the raman spectrum confirms the presence of the three bands of MWCNTs fingerprint, the D band a 1332 cm^−1^, the G band a 1576 cm^−1^ and the G' band around 2684 cm^−1^^46–48^. The D band indicates the degree of disarray on the CNT walls, the G band is sp^2^ and the G'-band refers to the inner and outer walls of MWCNTs^45,47,48^. In this composite, the first vibrations appear around 616 cm^−1^, when the surface is impregnated with HA through chemical effect (Fig. 10c), a significant shift to the right is noted, with the appearance of the first vibrations around 725 cm^−1^, which can be attributed to the vibrations of the narrowing of the C–C bonds^45–47^. As for the intensity of bands D (ID) and G (IG), it can be seen that the chemical effect led to a variation in the intensity of the bands. The ID/IG ratio, which refers to the density of defects present in the PLA/CNT-H composite, was lower when compared to the PLA/CNT, which presented ID/IG = 2,51. When surface impregnated with HA by chemical effect (Fig. 10c), the ID/IG ratio was 0.86. This result implies several sp^2^ domains and structural defects of the CNT network^44,46,47^. It is clear that the variation in the D and G band values, as well as the lower ID/IG intensity, imply an interaction between HA on the CNT surface, as well as possible structural transformation of PLA/CNT^44,45,47,48^.

### Mechanical test

#### Compression test

**Figure 11:**
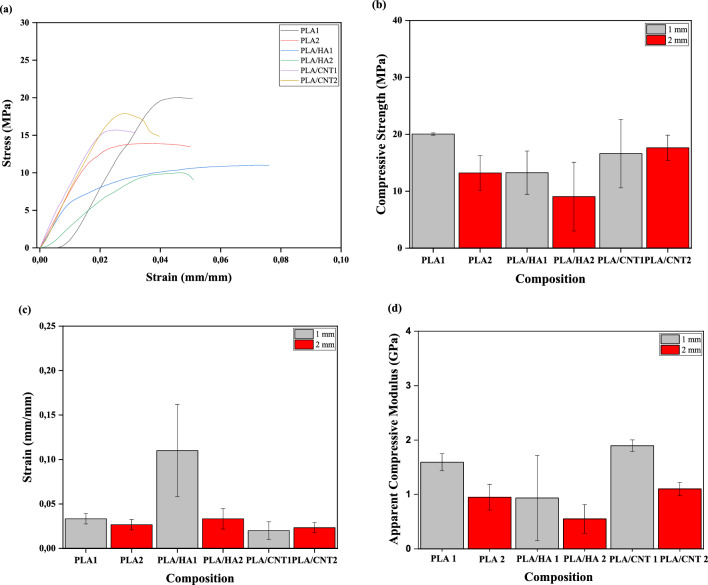
Mechanical properties obtained in uniaxial compression tests. The data represent the mean values and standard deviation of the mechanical properties of *scaffold*s with 1 and 2 mm squarebar boxes. (**a**) Stress–strain curves, (**b**) compressive strength, (**c**) strain and (**d**) apparent compressive modulus.

The compression test results are shown in Fig. 11. It is presented, in Fig. 11a, that the compositions studied disclose similar patterns of stress x strain, except PLA/HA 1 and PLA/HA 2 which show lower values of maximum stress, but greater strain, suggesting that HA induced a decrease in strength and consequent increase in the deformation capacity of the *scaffold*s.

Considering the compressive strength values of cancellous bone, which vary from 4 to 15 MPa^3,9,14,35^, the results presented in this study were satisfactory. In Fig. 11b, it can be seen that, among the compositions studied, pure PLA 1 shows the best compressive strength (20.02 MPa), followed by PLA/CNT2 (17.61 MPa) and PLA/CNT1 (16.62 MPa), suggesting that the addition of CNT increased the resistance of the materials studied. With regard to the addition of HA, the PLA/HA1 *scaffold* shows better mechanical performance than the PLA/HA2, suggesting that the addition of HA to the PLA and that the opening of squarebar boxes are factors that act in the decrease of the compressive strength *scaffold*. Figure 11c shows the behavior of the *scaffold*s concerning deformation, and it can be observed that the *scaffold*s with the highest deformation were PLA/HA1, followed by the PLA/HA2 and pure PLA. Figure 11d shows the graph of the apparent compression modulus and it can be seen that the *scaffold*s with the highest stiffness were PLA/CNT1 with 1.9 GPa and PLA1 with 1.59 GPa. According to^40–42^, the addition of CNT positively interferes with the mechanical properties of materials used as a support in bone tissue regeneration. With regard to the opening of squarebar boxes, it can be noted that, in general, the samples with an opening of 1.0 mm shows greater resistance to compression when compared to the samples with an opening of 2.0 mm, which proves, as already expected, that the opening of the squarebar boxes interferes in the mechanical properties of the material. Studies have shown that compressive strength decreases with increasing aperture size^13,14,35^. This result reveals that the addition of CNT to the PLA matrix led to a growth in the compressive strength by virtue of its intrinsic properties. The same pattern was also found by Vasconcelos et al.^37^ when they incorporated CNTs into PLA in the production of *scaffold*s for bone regeneration and obtained satisfactory results. PLA/CNT2 *scaffold* was the only one that presented better average resistance when compared to *scaffold*s with 1.0 mm opening. This may be associated with the insertion of CNT in PLA, which promotes an increase in the compressive strength. However, when comparing this property between the PLA/CNT1 and PLA/CNT2 *scaffold*s, considering the standard deviation, it is suggested that the squarebar box opening does not have a significant influence on this mechanical property. Furthermore, it can be observed that there is no significant difference between the evaluated compositions, suggesting that, mechanically, the HA and the CNT do not change the behavior of the *scaffold*s. This pattern might be associated with the type of geometry chosen for printing and the methodology of inverted immersion coating, which may not have contributed to the effective reinforcement of the PLA matrix by the HA and the CNT.

The results obtained in the compression test were by means of analysis of variance (ANOVA) displayed in Table 7. The ANOVA values for resistance show that the calculated F (2.72) is less than the critical F value (3.11). However, for deformation, the calculated F (6.93) is greater than the critical F value (3.11). For the apparent compression modulus, the calculated F (5.36) is also greater than the critical F value (3.11). With these results, it can be concluded that the averages for the properties of strain and apparent compression modulus are different, with a confidence index of 95%. Based on the results of the ANOVA, Tukey's test is necessary to investigate whether opening squarebar boxes can significantly interfere with *scaffold* properties. Table 8 shows Tukey´s test results. The minimum significant difference (m.s.d) value was used to evaluate which structure presented difference in the average values. For strain, m.s.d was calculated as 0.05 and for apparent compression modulus 0.86. Faced with this result, it was observed that the composition PLA2 show a significant difference from the composition PLA/HA2 and the composition PLA/HA1 show a significant difference from the compositions PLA1, PLA2, PLA/HA2, PLA/CNT1 and PLA/CNT2 indicating that opening the squarebar boxes modifies the strain values. Thereby, the PLA/HA1 *scaffolds* showed greater capacity for strain in compression than the other *scaffolds*. For the apparent compressive modulus, there was a significant difference between PLA 1 and PLA/HA1, PLA/HA2, between PLA 2 and PLA/CNT1, between PLA/HA1 and PLA/CNT1 and between PLA/HA2 and PLA/CNT1.

**Table 7:** Analysis of variance (ANOVA) of *scaffold*s printed by FDM.

Source	Sum of squares	Degrees of freedom	Mean of squares	F (calculated)	F critical	*p*-value
Compressive strength (MPa)						
Between the groups	229.12	5.00	45.82	2.72	3.11	0.07
Inside the group	202.52	12.00	16.88			
Total	431.64	17.00				
Strain (mm/mm)						
Between the groups	0.02	5.00	0.004	6.93	3.11	0.003
Inside the group	0.01	12.00	0.001			
Total	0.02	17.00				
Apparent compressive modulus (GPa)						
Between the groups	4.22	5	0.84	5.36	3.11	0.01
Inside the group	1.90	12	0.16			
Total	6.12	17

**Table 8:** Tukey test of pure PLA, PLA/HA and PLA/CNT *scaffold*s with 1 and 2 mm squarebar boxes.

	PLA1	PLA2	PLA/HA1	PLA/HA2	PLA/CNT1	PLA/CNT2
Strain (mm/mm)/m.s.d. = 0.05						
PLA1	0	0.01	**0.08**	0.00	0.01	0.01
PLA2	0.01	0	**0.08**	0.01	0.01	0.00
PLA/HA1	**0.08**	**0.08**	0	**0.08**	**0.09**	**0.09**
PLA/HA2	0.00	0.01	**0.08**	0	0.01	0.01
PLA/CNT1	0.01	0.01	**0.09**	0.01	0	0.00
PLA/CNT2	0.01	0.00	**0.09**	0.01	0.00	0
Apparent compressive modulus (GPa)/m.s.d. = 0.86						
PLA1	0	0.64	**0.95**	**1.04**	0.30	0.49
PLA2	0.64	0	0.30	0.40	**0.95**	0.15
PLA/HA1	**0.95**	0.30	0	0.09	**1.25**	0.46
PLA/HA2	**1.04**	0.40	0.09	0	**1.35**	0.55
PLA/CNT1	0.30	**0.95**	**1.25**	**1.35**	0	0.79
PLA/CNT2	0.49	0.15	0.46	0.55	0.79	0

Significant values are in bold.

### Cellular biocompatibility

#### Morphological analysis and cellular viability

**Figure 12:**
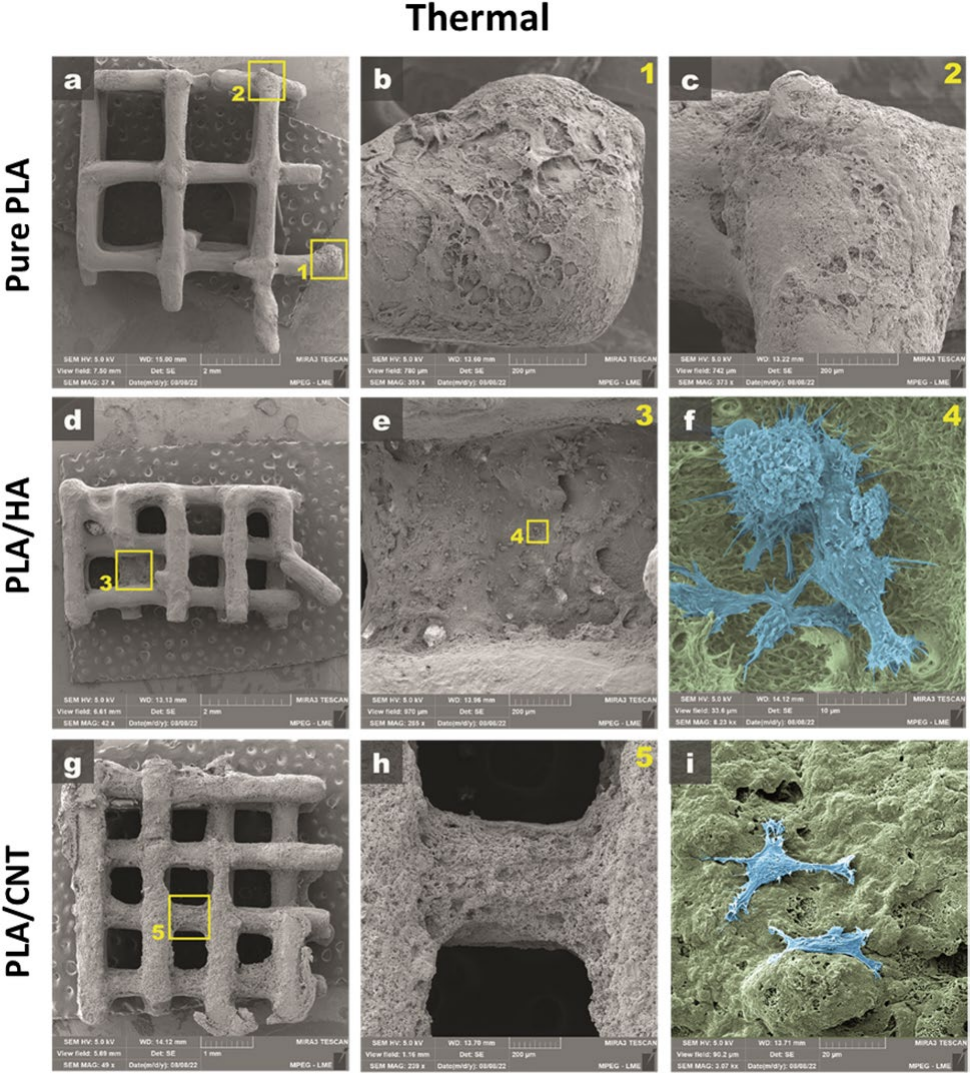
Scanning electron micrograph of pure PLA, PLA/HA and PLA/CNT *scaffold*s impregnated by thermal effect. (**a**–**c**) Pure PLA, observe *scaffold* coverage, but absence of adherent cells. (**b1**) Enlargement of area 1 highlighted in (**a**). (**c2**) Enlargement of area 2 highlighted in (**a**). (**d**–**f**) PLA/HA, observe *scaffold* surface covering (green-**f**), with the presence of adherent cells and emission of membrane extensions (blue-**f**). (**e3**) Enlargement of area 3 highlighted in (**d**). (**f4**) Magnification of area 4 highlighted in (**e**). (**g**–**i**) PLA/CNT, observe material coverage (green-**i**) and cell adhesion with emission of membrane extensions (blue-**i**). (**h5**) Enlargement of area 5 highlighted in (**g**).

**Figure 13:**
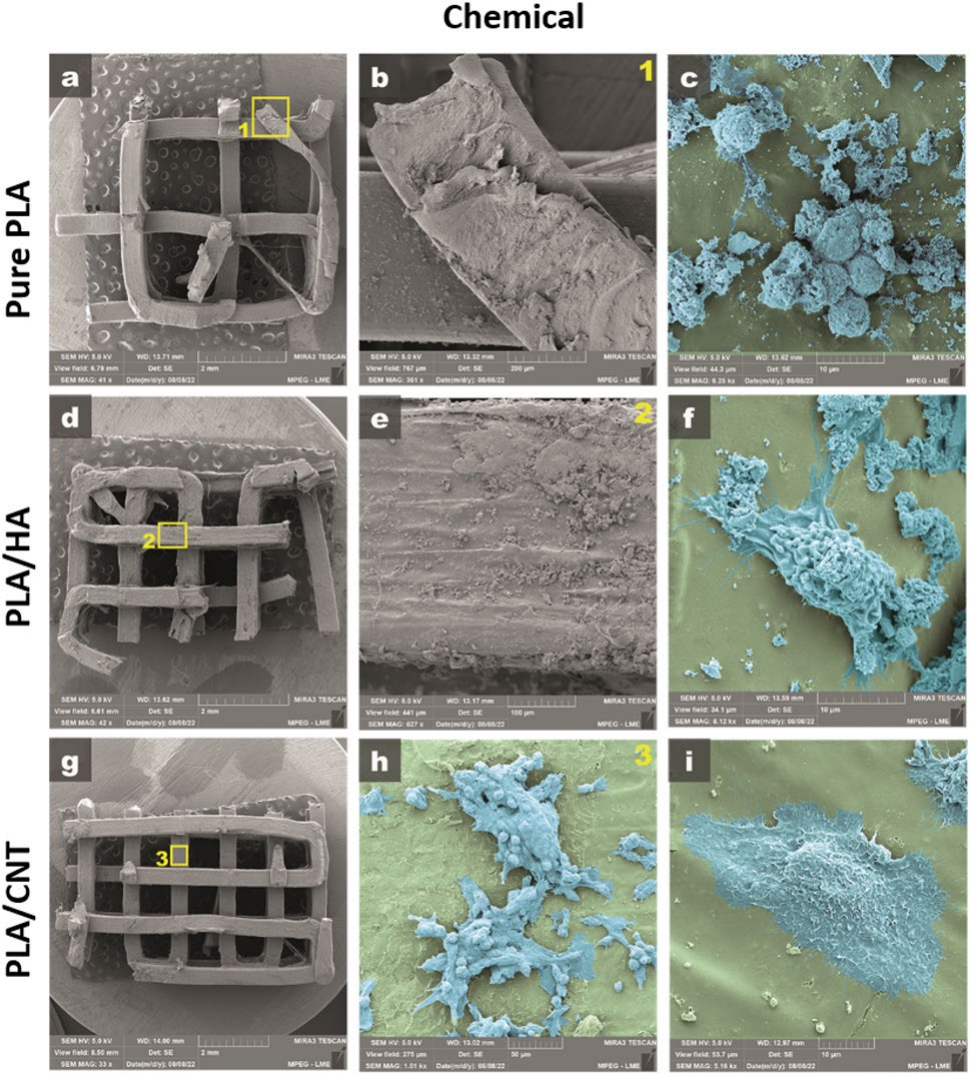
Scanning electron micrograph of pure PLA, PLA/HA and PLA/CNT *scaffold*s impregnated by chemical effect. (**a**–**c**) Pure PLA, observe *scaffold* coverage (**b**) and presence of cells with atypical morphology, characteristic of cells in the process of cell death (**c**). (**b1**) Enlargement of area 1 highlighted in (**a**). (**d**–**f**) PLA/HA, observe *scaffold* surface covering (**e**), with the presence of adherent cells, with the emission of membrane extensions, but also the presence of cellular debris (blue-**f**). (**e2**) Enlargement of area 2 highlighted in (**d**). (**g**–**i**) PLA/CNT, observe material coating (green-**h**), formation of cell aggregates (blue-**h**) and high cell adhesion with emission of membrane extensions (blue-**i**). (**h3**) Enlargement of area 3 highlighted in (**g**).

**Figure 14:**
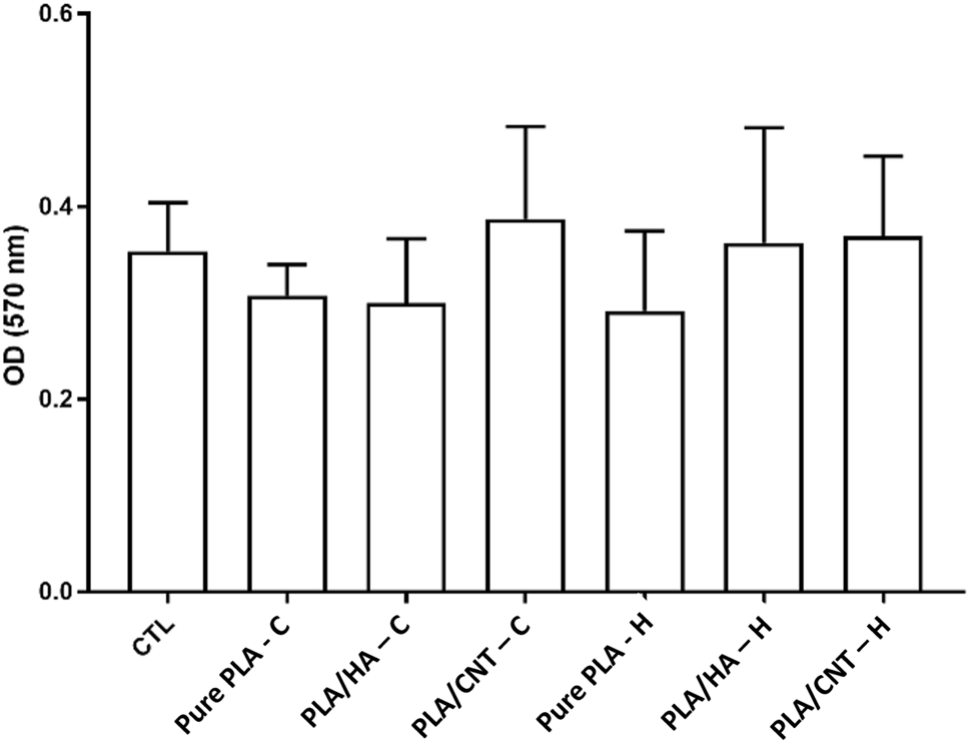
Cell viability graph of murine fibroblasts incubated with pure PLA, PLA/HA and PLA/CNT *scaffold*s and evaluated by the MTT method. There was no significant difference between the control group and the test groups (Student t-test, p > 0.05). H: impregnation by thermal effect; C: impregnation by chemical effect.

The analysis of the adhesion and morphology of the fibroblasts after 48 h of culture proved that the cells have better adhesion and spreading power in the PLA/HA and PLA/CNT *scaffolds*, for both the openings of the 1 and 2 mm squarebar boxes, in preparation methods, either by thermal-H (Fig. 12) or chemical-C (Fig. 13) effect. The PLA/HA-H *scaffold* (Fig. 12e,f) performed better than the PLA/HA-C (Fig. 13e,f) for both openings of the squarebar boxes, where it is possible to observe cells spread out (Fig. 13f), however, with many cell debris, similar to what was observed in the *scaffold* prepared only with pure PLA (Fig. 13c) for the two squarebar box openings. In contrast, PLA/CNT-H *scaffold* (Fig. 12h,i) and PLA/CNT-C (Fig. 13h,i) showed excellent cell adhesion and maintenance of typical cell morphology of fibroblasts, with spreading, adhesion and emission of cellular processes (Figs. 12i, 13h), such as phyllopodia and lamellipodia (Fig. 13i). These observations confirm the data observed in the cell viability tests. Fibroblasts incubated with the pure PLA, PLA/HA and PLA/CNT *scaffold*s showed high viability after evaluation by the MTT assay for both forms of preparation (impregnation by thermal effect —H and impregnation by chemical effect —C) and for the two openings of squarebar boxes. More than 85% of cells remained viable after 48 h of incubation with all *scaffolds* tested, and the groups that had the CNT in the composition showed the best results, both for thermal effect (more than 95% cell viability) and for chemical effect (acetone), as shown in Fig. 14. Fibroblasts are commonly used as an in vitro model to analyze the compatibility of various compounds and biomaterials^49–51^. These cells play a crucial role in bone tissue regeneration and come into contact with the *scaffold* during clinical application. Additionally, fibroblasts have demonstrated osteogenic properties by generating osteoblasts, making them a suitable in vitro model for investigating the *scaffold* in present study.

## Summary and conclusions

The development of *scaffold*s of poly (lactic acid) (PLA)/hydroxyapatite (HA)/carbon nanotubes (CNT) biocomposites for fibroblast cell proliferation by additive manufacturing shows that HA produced from precipitation technique displays high crystallinity and nanometric grain sizes. Despite the presence of CaO and non-stoichiometric HA, it was observed that it was possible to obtain the bioceramic at elevated temperatures, without significantly altering its biocompatibility. Furthermore, a characteristic HA morphology was observed with clusters similar to prismatic crystallites with hexagonal basal sections.

HA decreased the thermal stability of PLA, as well as the impregnation of HA on the surface of *scaffold*s by chemical effect, leading to a total mass loss of 97.87%, thus suggesting that the best technique for impregnating ceramics is thermal effect.

Although no statistical difference was observed between the compositions, with regard to compressive strength, PLA, PLA/CNT1 and PLA/CNT2 *scaffold*s showed the highest values for this property, surpassing the values of cancellous bone. Moreover, it was observed that squarebar boxes opening can influence the mechanical properties of strain and apparent compression modulus, with PLA/HA1 being the *scaffold* that presented the highest deformation capacity among the configurations studied. It is also suggested that the insertion of HA and CNT changes the apparent compression modulus due to structural changes in the PLA polymeric chain during the printing process, promoting greater rigidity between the chains.

Cellular biocompatibility tests revealed that more than 85% of the tested fibroblasts remained viable after 48 h of incubation, and the *scaffold*s containing CNT showed more than 95% of cell viability. Therefore, PLA/CNT1 and PLA/CNT2 *scaffold*s are viable alternatives for application in cell regeneration processes, as they combine desirable mechanical performance with excellent cell viability, and might be an alternative for future use in tissue engineering.

## Methods

The reagents used in this study are described in Table 9.

**Table 9:** Description of reagents used in this study.

Reagents	Purity (P.A)	Manufacturer	Quantities
Phosphoric acid (H_3_PO_4_)	85%	VETEC	160 mL
Calcium hydroxide (Ca(OH)_2_)	X	ISOFAR	50 g
Ammonium hydroxide (NH_4_OH)	30%	NEON	90 mL
Acetone ((CH_3_)_2_CO)	X	ISOFAR	5 mL

### Hydroxyapatite (HA) synthesis

HA powder was obtained by the acid–base reaction from a suspension of 50 g of calcium hydroxide powder (Ca(OH)_2_) along with the addition of 160 mL of phosphoric acid solution (H_3_PO_4_) at a concentration of 1.0 M. The ambient temperature was maintained constant, as well as the pH of the experiment, which was kept at 10 by the controlled addition of 90 mL of ammonium hydroxide (NH_4_OH), also at a concentration of 1.0 M.

The magnetic stirrer, brand TELGA, was used for complete homogenization between the calcium hydroxide powder and the phosphoric acid solution. The solution was stirred for 2 h, then left to stand for another 24 h. Subsequently, the product obtained was washed with distilled water and filtered under vacuum, where the resulting powder was dried in an oven of the brand Gigante model G42L at 100 °C for 24 h.

**Figure 15:**
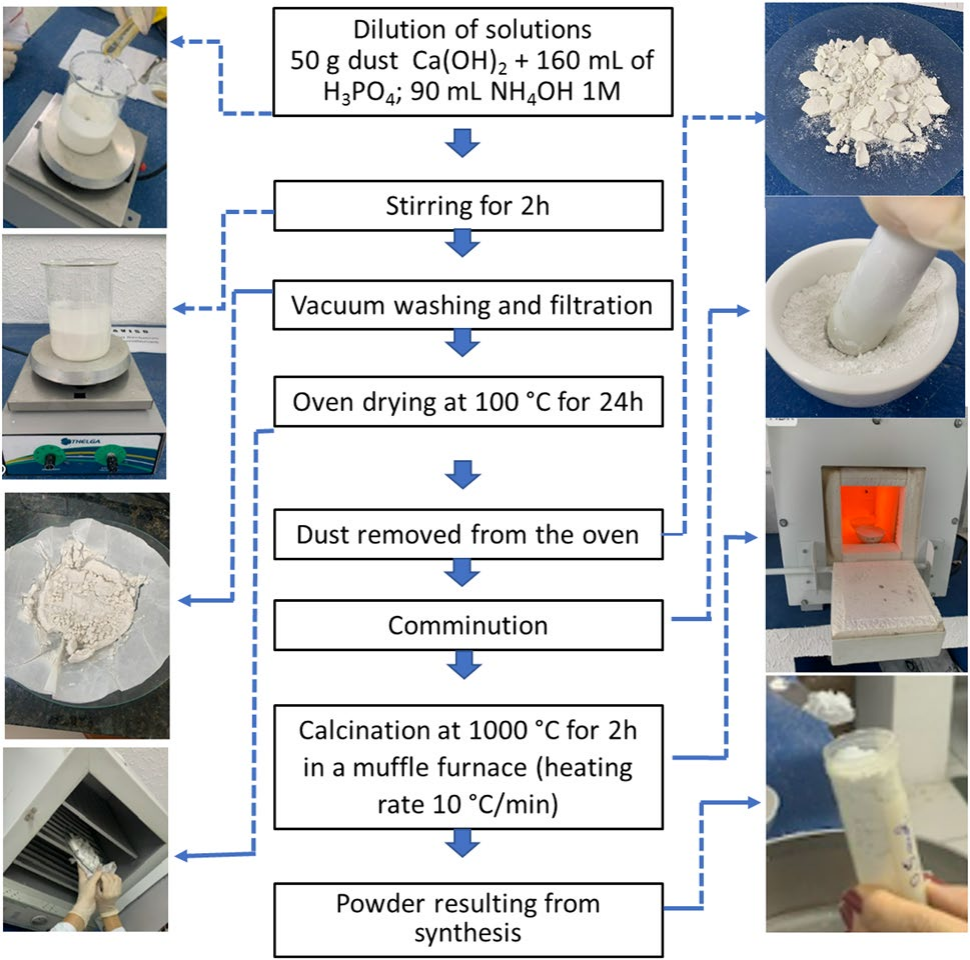
Process of synthesis of HA powder.

After the drying stage, the powder was ground and calcined at a temperature of 1000 °C for 2 h, in a muffle furnace brand SolidSteel SSFm—6.7 L, with a heating rate of 10 °C/min. The cooling occurred gradually and the sample remained inside the furnace until it reached ambient temperature. After calcination, the product went through a new comminution stage, and sieved through a sieve with an aperture of 200 mesh. Figure 15 shows the flowchart of the synthesis process of the HA powder.

### Preparation of the composite PLA/HA and PLA/CNT filaments and production of biocomposites by 3D printing

The filament of PLA used in the preparation of the composites was purchased from the company 3D Fila. Table 10 shows the physical characteristics of PLA according to the manufacturer.

**Table 10:** Physical characteristics of the PLA.

Density	1.08 g/cm^3^
Glass transition temperature	54 °C
Melting temperature	180 °C
Diameter	1.75 mm

**Figure 16:**
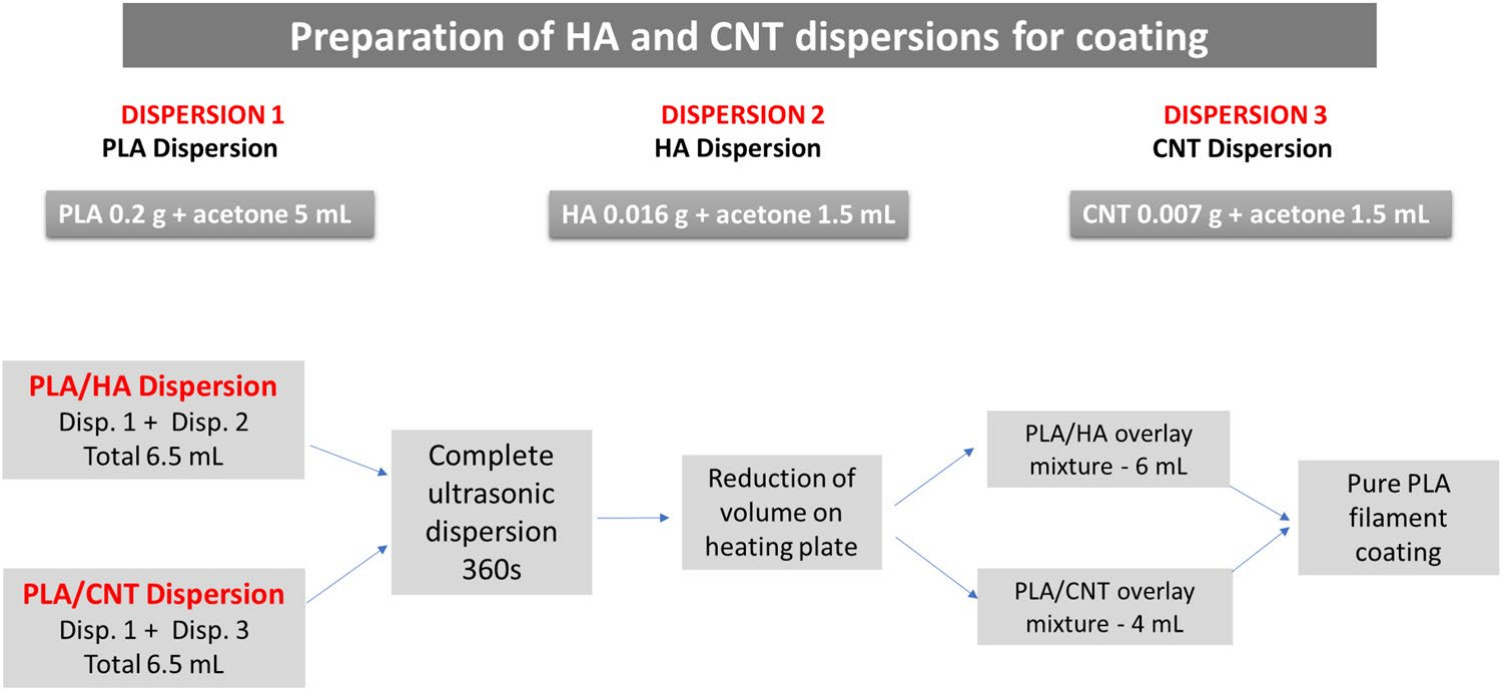
Production process of dispersions for covering 0.7 mm of pure PLA filament.

The multi-walled carbon nanotubes (MWCNT), synthesized by chemical vapor deposition (CVD), were donated by the 3D Nanostructuring Laboratory of the Federal University of Pará, Brazil. The preparation of composite filaments made of poly (lactic acid) and hydroxyapatite (PLA/HA) and poly (lactic acid) and carbon nanotubes (PLA/CNT) followed the same steps. 0.2 g of PLA filament was used, cut into pieces, and dispersed in 5 mL of acetone, at ambient temperature. Then they were mixed with the HA (0.016 g) and CNT (0.007 g) dispersed in 1.5 mL of acetone to produce the coating mixture, as shown in Fig. 16.

Figure 16 shows the production process of PLA/HA and PLA/CNT dispersions, respectively, for covering pure PLA filament.

### Coating process with the PLA/HA and PLA/CNT mixtures

To cover the pure PLA filaments with the PLA/HA and PLA/CNT mixtures, a funnel with an opening at the lower end with a diameter close to the diameter of the pure PLA filament was used. Firstly, PLA/HA and PLA/CNT dispersions were added separately to the funnel, then, 0.7 m of pure PLA filament was inserted through the bottom end of the funnel, directing upwards until the entire filament was covered (inverted immersion) with a pulling rate of 5 cm/s. Then, the coated filament was dried and adhered at ambient temperature (~ 30 °C) for 2 h. This process was repeated for the coating of the second layer of the dispersions. At the end of the second coating step, the coated filaments were taken to dry for another 5 h. After that, the filaments coated with PLA/HA and PLA/CNT were taken to 3D printing.

**Figure 17:**
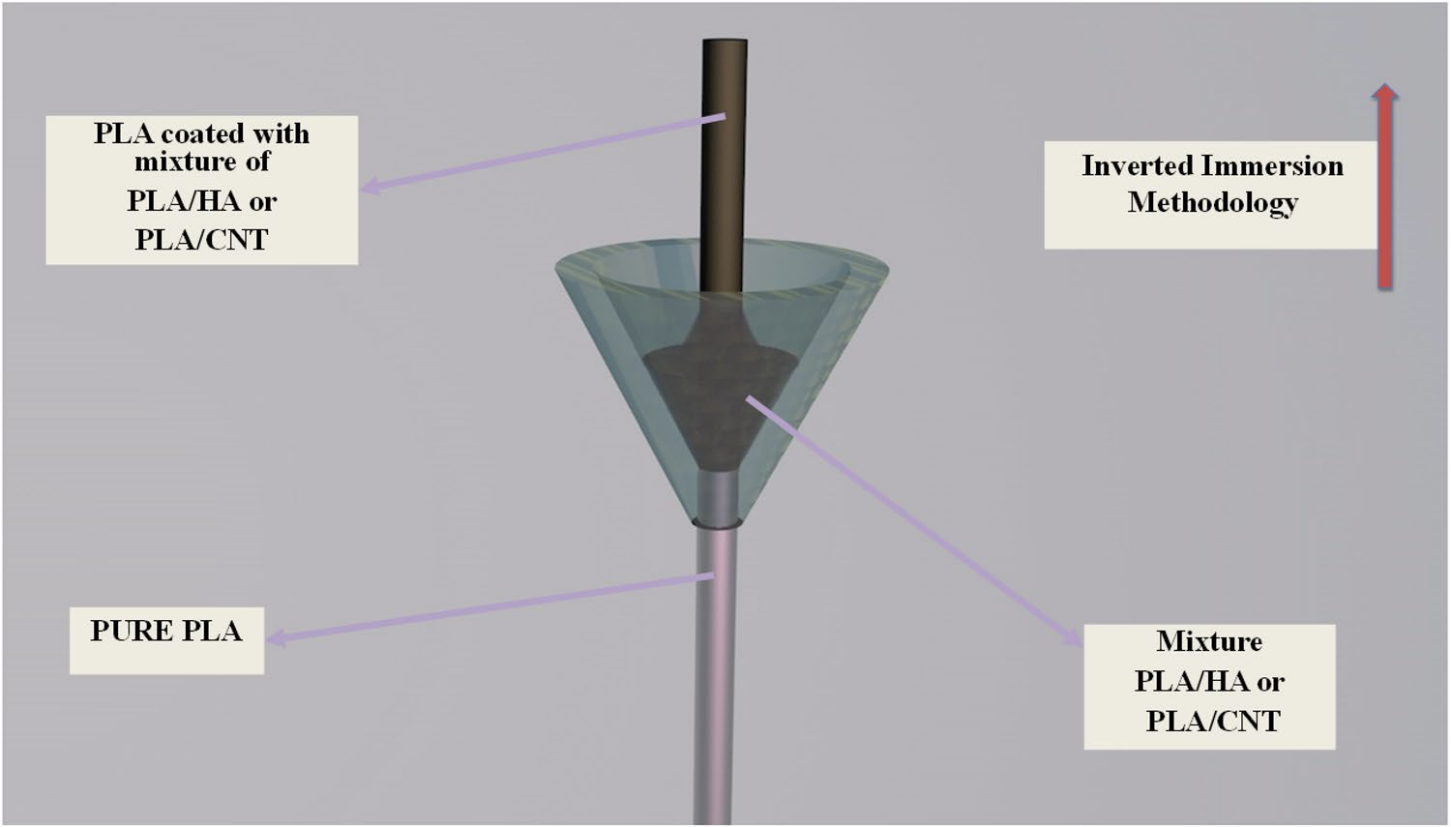
Methodology of inverted immersion of PLA coated with PLA/HA or PLA/CNT mixture.

The process of covering the pure PLA filament with the PLA/HA and PLA/CNT dispersions by inverted immersion can be seen in Fig. 17.

### 3D printing of *scaffold*

**Figure 18:**
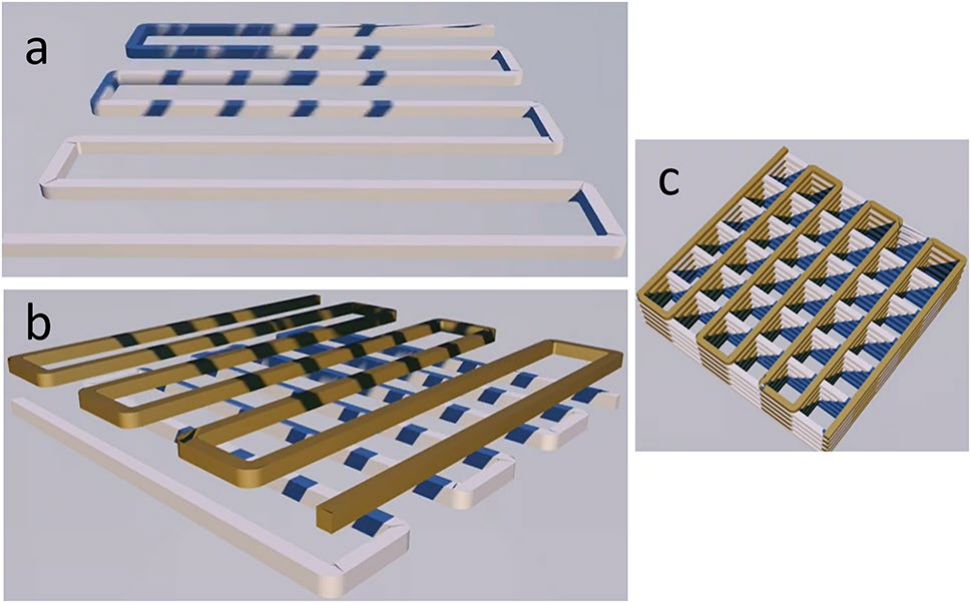
Projection of serpentine-shaped *scaffold*s, interspersed layer by layer. In (**a**), the first layer is observed on a given axis. In (**b**), the second layer is superimposed vertically. In (**c**), overlapping layers forming *scaffold*s.

**Figure 19:**
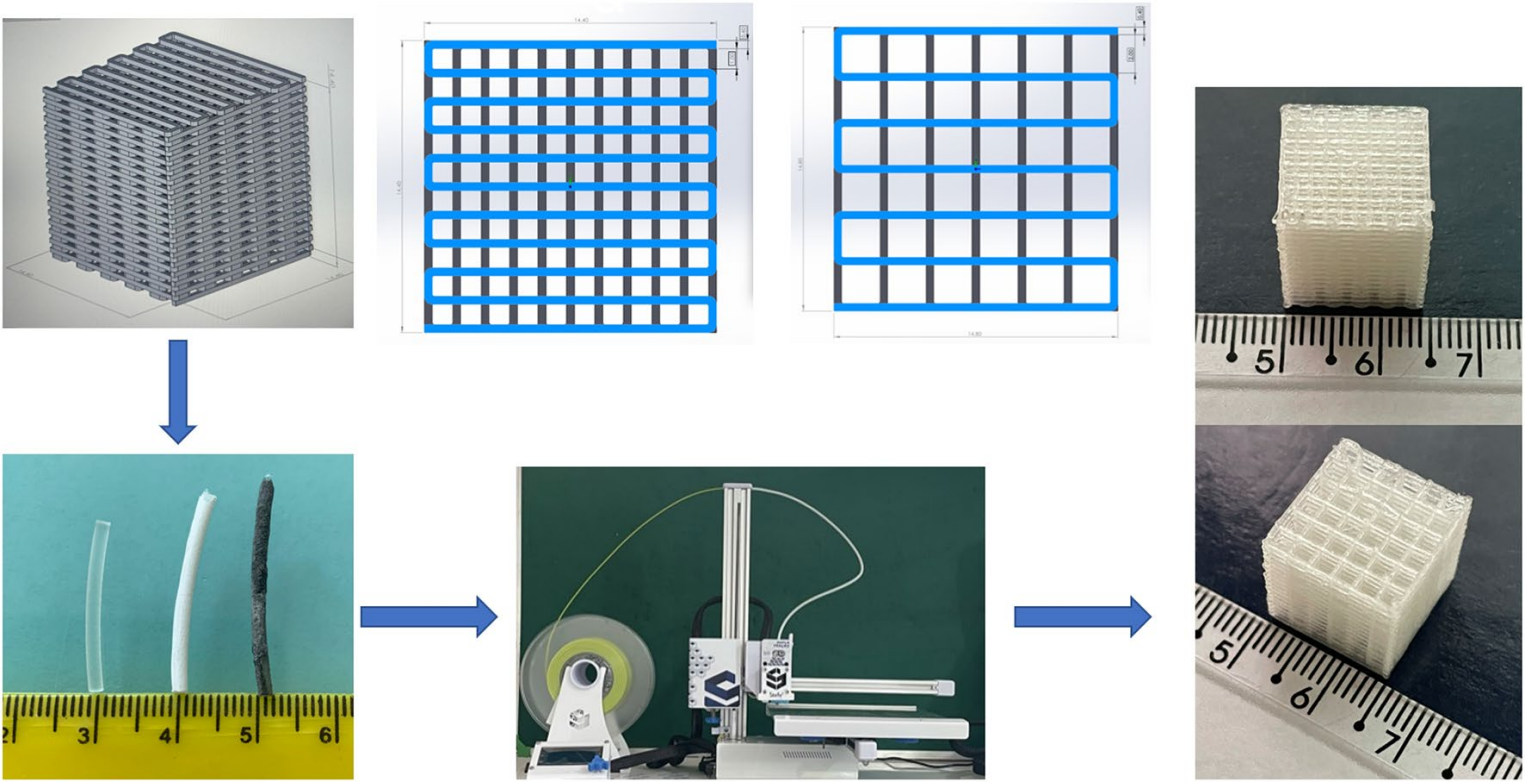
Process of obtaining *scaffold*s by FDM printing. The *scaffold*s were designed with 1 and 2 mm squarebar boxes by the modeling software^52^. PLA filaments coated with HA and CNT were subjected to a 3D printer to produce *scaffold*s printed by FDM.

This study produced by 3D printing, six *scaffold* samples, two pure PLA, two PLA/HA and two PLA/CNT where, for each type of *scaffold*, squarebar boxes were 3D printed with openings varying from 1.0 to 2.0 mm. After having been covered with PLA/HA and PLA/CNT mixture, PLA filaments were prepared for 3D printing using the fused deposition modeling (FDM), where the material is usually melted shortly after the glass transition temperature and is then extruded in a pattern close to or above the previous extrusions, creating a layer-by-layer object illustrated in Fig. 18. The print patterns of the *scaffolds* were designed in the form of serpentines, arranged in an interleaved way layer by layer, initially vertically, then rotating it horizontally by 90 degrees to create the next layer. The first layer is observed on a certain axis and the second layer is superimposed vertically. This process is repeated successive times until the *scaffold* is formed. The temperature of the initial layer was 215 °C and the temperature of the following layers was 195 °C. Firstly, the *scaffolds* were modeled using Solidworks® software^52^, using Prusa-Slicer® slicer, designing 14.40 mm high and 14.80 mm wide *scaffolds*, with 1.0 and 2.0 mm squarebar boxes respectively, as shown in Fig. 19.

At the end of the 3D printing, six distinct *scaffolds* were generated: 3 *scaffold*s with 1.0 mm squarebar boxes (1 of pure PLA, 1 of PLA/HA and 1 of PLA/CNT) and 3 *scaffold*s with 2.0 mm squarebar boxes (1 of pure PLA, 1 of PLA/HA and 1 of PLA/CNT). The equipment used to print the *scaffold*s was a commercial Stella brand 3D printer, model lite 3, of the company Boa Impressão 3D, Curitiba-PR, Brazil. The parameters used in the 3D printing of the *scaffolds* are described in Table 11.

**Table 11:** 3D printing parameters.

Parameters	Specification
Filament diameter	1.75 mm
Tip diameter	0.4 mm
Tip initial temperature	215 °C
Initial temperature of the first layer	215 °C
Table temperature: other layers	195 °C
Height of layers	0.1 mm per layer
Wall thickness	0.4 mm
Speed (print rate)	20 mm/s

### Impregnation with HA powder on the *scaffold* surface

To investigate the interference of HA in the biological properties of *scaffold*s, impregnation of HA powder on their surface was performed using two different methodologies: impregnation by thermal effect (PLA/CNT-H) and impregnation by chemical effect by immersion in a solution of HA diluted in acetone PA (PLA/CNT-C).

For impregnation by thermal effect (H), the samples were conditioned separately in petri dishes where the HA powder was deposited on them. Then, they were placed in a muffle furnace with an initial temperature of 10 °C until reaching 150 °C, with a heating rate (TxA) of 6 °C/min. The TxA was then increased to 9 °C/min until reaching a temperature of 180 °C, the melting temperature of PLA^10,12,17^.

In the impregnation by chemical effect (C), 0.016 g of HA powder was diluted in 1.5 mL of acetone to PA, keeping the solution in an ultrasonic shaker for 360 s for complete solubilization. Taking into account the melting time of PLA in acetone, approximately 17 s, each sample fragment was then immersed separately in the HA solution for 15 s. They were then dried in an oven at 100 °C for 10 min and then weighed to evaluate mass gain as described in Table 3.

### Microstructural characterization of the hydroxyapatite powder and the PLA/HA and PLA/CNT composites

The HA powder was characterized by XRD, FTIR, SEM and EDS. For pure PLA, PLA/HA and PLA/CNT composites, TGA was performed as well as SEM, optical microscopy, Raman spectroscopy and cell viability test.

### X-ray diffraction (XRD) of HA powder

XRD analyzes were performed using an X'Pert Pro 3 MPD (PW 3040/60) Panalytical X-ray diffractometer, with PW3050/60(θ-θ) goniometer and with Cu anode ceramic X-ray tube (Kα1 = 1.540598 Å) model PW3373/00, long fine focus. The XRD patterns were compared according to the standard catalog database present in the fixed indexes in JPDS—PDF—2 present in the ICSD (world's largest database for inorganic crystalline structures), analyzed in the X'pert High Score Plus software^53^. To quantify the phases present, the Rietveld refinement was performed using Material Analysis Using Diffraction (MAUD) free software^54^.

### Fourier transform infrared spectroscopy (FTIR) of the HA powder

The FTIR analysis was performed in an equipment Bruker, model Vertex 70 v (Madison, WI, USA). The samples were analyzed in the middle infrared (MIR) spectral region, from 4000 to 400 cm^−1^, at 100 scans and a resolution of 8 cm^−1^.

### Scanning electron microscopy (SEM)

SEM was performed to analyze the HA powder, the pure PLA, PLA/HA and PLA/CNT *scaffold*s and after the cell adhesion testing on the *scaffold*s. For the HA powder and *scaffold*s before the cell adhesion testing, the samples were metalized with a 10 to 15 nm medium-thickness gold film. For samples after the adhesion test, they were fixed with 2.5% glutaraldehyde (25%), 4% formaldehyde (10%), in 0.1 M sodium cacodylate buffer with pH 7.2, post-fixation in 1% osmium tetroxide and 0.8% potassium ferrocyanide and dehydrated in ethanol (Merck). The critical point drying technique is the final stage of material preparation for SEM analysis. Finally, they were metalized as already described. All analyzes were performed in a TESCAN scanning electron microscope, model Mira3, with a secondary electron beam.

### Energy dispersive spectroscopy (EDS) of the HA powder

Samples for EDS were metalized with carbon and the analyzes were carried out at working distances of 15 mm and 15 kV. It was used the Oxford brand EDS detector model X-act.

### Optical microscopy of *scaffolds*

The pure PLA, PLA/HA and PLA/CNT *scaffolds* were subjected to optical microscopy through the Even microscope, model MDA1300.

### Thermogravimetric analysis (TGA) of the *scaffolds*

To assess the thermal stability of the material, TGA was performed on pure PLA and on PLA/HA composites; PLA/CNT and PLA/CNT impregnated with HA powder on the material surface by thermal and chemical effect. TGA was carried out using NETZSCH equipment, model STA 449 F3 Jupiter, with temperature variation in the range from 20 to 800 °C and a heating rate of 10 °C/min, in a nitrogen atmosphere.

### Raman spectroscopy of HA powder and *scaffolds*

Raman spectroscopy was performed on a LabRam spectrometer, model HR EVOLUTION, HORIBA brand, for the measurements, the following conditions were used: laser: 633 nm; range: 600–3200 cm^−1^, except for the HA sample, which used a range of 100–3200 cm^−1^ because the sample showed bands from 100 cm^−1^. The run time was 120 s with accumulations of 2 s, with the laser power filter at 5% and the lens at 100x.

### Compression test

**Figure 20:**
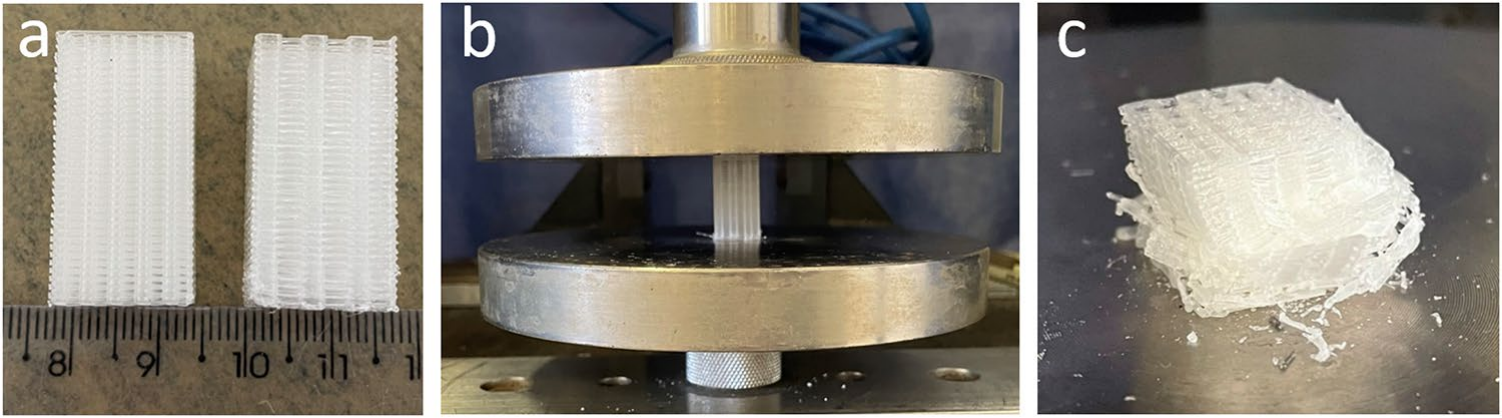
Compression test. (**a**) Specimen; (**b**) compression test; (**c**) specimen after the test.

Compression tests were performed in accordance with ASTM-D695-10^55^. To meet the specifications established by the standard, the specimens were made with dimensions meeting the requirement that the height be twice the size of the width. A set of test specimens of six samples for each material was used, totaling eighteen samples, with dimensions of 14.80 × 14.80 × 28 mm. The test was performed using the EMIC LINHA DL equipment, with a 500 kgf load cell, at a constant rate of 1 mm/min. The steps of the compression test are shown in Fig. 20. It is important to emphasize that the specimens used for the compression test were not impregnated with HA on the surface. Compressive strength and creep properties were calculated from the load displacement data. The apparent compression modulus was based on the slope of the stress–strain curve in the elastic region.

### Biocompatibility assessment

#### Cell cultivation and maintenance

Murine fibroblasts of the BALB/c 3T3 lineage, clone A31, from the Cell Bank of Rio de Janeiro, Brazil (BCRJ: 0047) were cultured at a density of 5 × 10^4^ cells/mL in Dulbecco's Modified Eagle Medium (DMEM) supplemented with 10% fetal bovine serum and kept in an oven at 37 °C in an atmosphere of 5% CO_2_ until reaching 80% confluency for later use.

#### Cell viability assessment

In 24-well plates, the *scaffold*s were added followed by the addition of 1 × 10^4^ cells/mL over the *scaffolds*. Plates containing cells and *scaffold*s were incubated for 48 h under the same conditions described previously. After this period, the supernatant was removed and 0.5 mg/mL MTT ([3-(4,5-dimethylthiazol-2-yl)-2,5-diphenyl tetrazolium bromide]) diluted in saline phosphate solution was added (phosphate buffered salin-PBS) and incubated for 3 h. Then, 200 μL dimethylsulfoxide (DMSO) were added to each well to solubilize formazan crystals and the plates were stirred for 10 min. The resulting solution was read in a spectrophotometer (BIO-RAD Model 450 Microplate Reader) at a wavelength of 570 nm^56^. As a testing control, cells were killed with a 10% formalin solution in PBS and as a control for cell viability, another group of cells were grown without the presence of *scaffolds*. The result was expressed considering the obtained optical density (optical density-OD-570 nm). For each type of *scaffold*, two independent experiments were performed in triplicate. Statistical analysis was performed using the GraphPad Prism 6™ software^57^, Student's t-test, with p < 0.05 being considered a statistically significant difference.

#### Morphological evaluation

Fibroblasts were cultivated as previously described and, later, they were cultivated with different types of *scaffold*s for 48 h. Thereafter, the samples were fixed in a solution containing 2.5% of glutaraldehyde, 4% of formaldehyde in 0.1 M sodium cacodylate buffer, pH 7.2 for 1 h. Afterward, the samples were post-fixed in a solution containing 1% osmium tetroxide and 0.8% potassium ferrocyanide for 1 h. The samples were then subjected to increasing dehydration in ethanol and, subsequently, critical point drying was performed^58^. Finally, the samples were metalized with a thin layer of gold and analyzed using a TESCAN scanning electron microscope, model Mira3.

## Data Availability

The datasets generated and/or analysed during the current study are not publicly available due be in the process of filing a patent but are available from the corresponding author on reasonable request.
